# Characterization of symbiotic and nitrogen fixing bacteria

**DOI:** 10.1186/s13568-022-01441-7

**Published:** 2022-07-30

**Authors:** Fanuel Kawaka

**Affiliations:** Department of Biological Sciences, Jaramogi Oginga Odinga University of Science and Technology, P.O. Box 210-40601, Bondo, Kenya

**Keywords:** Nodules, Characterization, Isolation, Nitrogen, Technique

## Abstract

Symbiotic nitrogen fixing bacteria comprise of diverse species associated with the root nodules of leguminous plants. Using an appropriate taxonomic method to confirm the identity of superior and elite strains to fix nitrogen in legume crops can improve sustainable global food and nutrition security. The current review describes taxonomic methods preferred and commonly used to characterize symbiotic bacteria in the rhizosphere. Peer reviewed, published and unpublished articles on techniques used for detection, classification and identification of symbiotic bacteria were evaluated by exploring their advantages and limitations. The findings showed that phenotypic and cultural techniques are still affordable and remain the primary basis of species classification despite their challenges. Development of new, robust and informative taxonomic techniques has really improved characterization and identification of symbiotic bacteria and discovery of novel and new species that are effective in biological nitrogen fixation (BNF) in diverse conditions and environments.

## Introduction

The process of biological nitrogen fixation (BNF) is catalyzed by a two-component nitrogenase complex (Yan et al. 2010). The enzyme nitrogenase catalyzes the simultaneous reduction of one N_2_ and 2 H + to ammonia and a molecule of hydrogen gas. The enzyme consists of two proteins, an iron protein and a molybdenum-iron protein. The entire process uses 16 mol of ATP and a supply of electrons and protons and occurs optimally between legumes and bacteria (de Carvalho et al. 2011).

$${\text{N}}_{2} + 8{\text{H}}^{ + } + 8{\text{e}}^{ - } + 16{\text{ATP}} \to 2{\text{NH}}_{3} + {\text{H}}_{2} + 16{\text{ADP}} + 16{\text{P}}_{{\text{i}}}$$

Symbiotic relationship between the roots of legumes and certain soil bacteria accounts for the development of a specific organ, the symbiotic root-nodule, whose primary function is nitrogen fixation (Shvaleva et al. 2010). Depending on the type of microorganism, the energy required for the reduction during N fixation is generated by photosynthesis, respiration or fermentation.

High rate of O_2_-respiration is necessary to supply the energy demands of the N reduction process however O_2_ also irreversibly inactivates the nitrogenase complex. These conflicting demands are met by control of O_2_ flux through a diffusion barrier in the cortex of nodules, which limits permeability to O_2_ (Matthay et al. 2019). Oxygen is then delivered to the bacteroids by the plant O_2_-carrier, leghemoglobin found in the nodule (Jones et al. 2007). To maintain the low-ambient O_2_-concentration within the nodule, N_2_-fixing bacteroids use a high-affinity cytochrome *cbb*_3_-type oxidase encoded by the *fixNOQP* operon to produce ATP (Pitcher and Watmough 2004).

There are diverse group of symbiotic bacteria found within the roots of legume plants. However, defining the identity of these closely related bacterial species remain a significant and challenging feature among taxonomists. Classification and taxonomy of bacteria nodulating legume plants have significantly changed in the last 30 years. Initially, classification and identification techniques relied mainly on biochemical, nutritional and serological characteristics together with host ranges. Currently, modern molecular tools and techniques have considerably improved the identification of legume nodulating bacteria. Molecular genetic markers are more sensitive and accurate in distinguishing closely related bacterial species and detect higher diversity compared to phenotypic methods. Traits used in phenotypic characterization include colony morphology, physiology or biochemical reactions of bacteria may vary based on the media and laboratory conditions. Nonetheless, all the taxonomic methods and techniques have their own weaknesses in studying the diversity and phylogeny of bacteria (Pontes et al. 2007). Studies have demonstrated that accurate identification of bacteria fixing nitrogen in legumes is vital for researchers in applied research and industry particularly those strains with high nitrogen-fixation ability (Franco-Duarte et al. 2019). The current review highlights selected common methods of detecting and identifying symbiotic bacteria in legume crops for improvement of nodulation efficiency and present their advantages and limitations.

## Phenotypic, cultural and metabolic characteristics

A wide range of morphological, cultural and metabolic characteristics are used to describe and identify nodule bacteria. Phenotypic traits often used include growth rate, mucous production, colony characteristics, and change in pH of the isolates during growth on Yeast Extract Mannitol Agar (YEMA) media (Hungria and Kaschuk 2014; Maatallah et al. 2002). Growth in YEMA has been used to classify pure bacterial colonies into either slow or fast growers, presence or absence of mucous, low or high pH among other properties such colony colour, margin and diameter (Kawaka and Muoma 2020). Microscopy and staining also group isolates into either Gram positive or negative in addition to absorption of Congo red dye (Kawaka et al., 2014). In a study conducted in western Kenya, Kawaka et al. (2016) highlighted phenotypic and cultural characteristics that are commonly used to describe native symbiotic isolates from different soils (Table 1). As indicated in Table 1, the pure bacterial isolates are mostly characterized to give presumptive identity, establish relationships between isolates and to understand their behavior.

**Table 1:** Morphological and cultural characteristics of indigenous symbiotic bacterial isolates from legumes

Characteristics	Morpho-cultural description of isolates											
	*KSM 1*	*KSM 2*	*KSM3*	*KSM 4*	*KSM 5*	*KSM 006*	*KSM 7*	*KSM 8*	*MMUST 3*	*MMUST 4*	*MMUST 5*	*MMUST 6*
Congo Red Absorption	✓	✓	✓	✓	✓	✓	✓	✓	✓	✓	✓	✓
BTB reaction	✓	✓	✓	✓	✓	✓	✓	✓	✓	✓	✓	✓
Colony colour	Cream yellow	Cream White	Cream white	Milky white	Milky white	Cream yellow	Cream white	Cream white	Milky white	Cream yellow	Milky white	Cream yellow
Colony transparency	Opaque	Translucent	Opaque	Opaque	Translucent	Translucent	Opaque	Translucent	Opaque	Translucent	Opaque	Opaque
Colony appearance	SHINY	SHINY	SHINY	DULL	DULL	SHINY	SHINY	SHINY	SHINY	SHINY	DULL	DULL
EPS production	✓	✓	✓	x	x	✓	✓	✓	x	✓	x	✓
Colony texture	Firm dry	Smooth viscous	Smooth viscous	Firm dry	Firm dry	Smooth viscous	Smooth viscous	Smooth viscous	Smooth viscous	Smooth viscous	Firm dry	Firm dry
Colony shape	Circular	Oval	Oval	Circular	Circular	Circular	Oval	Oval	Circular	Circular	Circular	Circular
Colony elevation	Convex	Convex	Convex	Convex	Convex	Convex	Convex	Convex	Convex	Convex	Convex	Convex
Colony diameter (mm)	3.7	4.7	5.7	3.7	4.0	3.7	5.0	3.3	4.7	3.3	3.0	1.0
Gram stain	✓	✓	✓	✓	✓	✓	✓	✓	✓	✓	✓	✓
Colony margin	Entire	Entire	Entire	Entire	Entire	Entire	Entire	Entire	Entire	Entire	Entire	Entire

*BTB* bromothymol blue, *EPS* exopolysaccharides, ✓ positive reaction, ×  negative

Additional methods such as cell protein banding pattern, multilocus enzyme electrophoresis and tolerance to stress, salinity, heavy metals and high temperatures may be used to characterize nodule bacteria (Dekak et al. 2018). These tests were suggested as a way to resolve taxonomic difficulties but were later considered to be impracticable (Graham and Parker 1964). Despite the criticism, phenotypic, cultural and metabolic methods are frequently carried out in combination with other techniques and provides the primary basis for species classification (Li et al. 2020).

## Cross inoculation

The concept of cross inoculation depends on the symbiotic bacteria ability to selectively form nodules with a group of legume hosts (Mendoza-Suárez et al. 2020). Nodulated bacterial strains are described as being specific when they are selective in their host range and considered promiscuous when they have a broader range of host (Kawaka 2016; Provorov et al. 2013). However, several researches have reported that legumes are nodulated with bacteria that are not within their own groups (Pankievicz et al. 2019). The cross inoculation method like other earlier methods of identification does not take into consideration the nitrogen fixation abilities of the bacteria. Studies have reported that bacterial strains form nodules on leguminous hosts but only a few of the species can effectively fix nitrogen on those host plants (Bourion et al. 2018). Consequently, the use of cross inoculation technique in the classification of nodule bacteria has reduced due to associated setbacks.

Traditionally, classification of symbiotic and nitrogen fixing bacteria was based on cross-inoculation concept that depended largely on the degree of host specificity. It is therefore important that such a classification requires a standardization of nodulation tests and the control of optimal conditions for plant growth. The genes involved in the development of the symbiotic organ in plant roots, stems or nodule are collectively called nodulation (*nod*) genes. These genes are unique to symbiotic bacteria and the phylogenies of *nodA*, *nodB*, *nodC* and *nodD* resemble each other but vary considerably from the phylogeny of 16S rRNA (Aguilar et al. 2022). Studies have indicated that the phylogenies of *nod* genes may correlate with the host plant (Aguilar et al. 2022; Mohammed et al. 2018). For example, as a nodulation gene marker, nodC gene is a common nod gene essential for nodulation in all symbiotic bacterial species. Laboratory analyses performed by using a variety of techniques showed various degrees of correlation between symbiotic genes and chromosomal genotypes. Generally, these findings concluded that symbiotic genes appear to have been transferred between strains (Laranjo et al. 2014). Symbiotic bacterial genes are usually located on plasmids thus increasing their likelihood of gene transfer. In contrast, *nif* genes are found in many bacteria besides those fixing nitrogen however it remains unclear whether these genes are evolutionary part of the symbiotic genome or part of the “normal” bacterial genome (Drew et al. 2021). Different authors have reported that the phylogeny of *nifH* closely resembles that of 16S rRNA genes and that these genes probably share a common evolutionary history (Drew et al. 2021; Watanabe and Horiike 2021). However, there is also evidence of phylogenetic discordance that could be due to lateral transfer of *nif* genes (Lau et al. 2014). Due to convenience and agronomic significance in selecting strains with the potential use as inoculants for particular legumes, many researchers continue to justify the use of this method (Gopalakrishnan et al. 2015).

## Serology

The use of comparative serology provides valuable information about relationships between prokaryotes and has been helpful for rapid identification of various species of bacteria (Fair and Tor 2014). The technique differs from other standard procedures only in the preparation of antigens, but it is less time-consuming (Solomon et al. 2012). The technique involves the use of antibodies raised against surface antigens of the test strain to detect the presence (or absence) of that strain in a suspension through agglutination, immunodiffusion, immunofluorescence or the enzyme-linked immunosorbent assay (ELISA) (Maurin 2020).

Since the antigenic properties of the nodule bacteria are stable characteristics, the method is particularly useful in ecological studies as it does not modify the strain or alter its nodulation competitiveness (Spriggs and Dakora 2009). The immunofluorescence technique has also been successfully used to rapidly identify rhizobial strains, though this requires expensive equipment and large quantities of labelled antibody (Spriggs and Dakora 2009).

Serological method relies on the reaction of antigen and antibody to assess symbiotic bacterial diversity in the rhizospheric microbiome. Serological studies focusing on indigenous nodule based bacteria demonstrate significant strain variations within and among different geographic regions (Stępkowski et al. 2018). The use of serology has made it possible to relate the occurrence of particular serogroups in a particular location to certain soil parameters like pH or total nitrogen content (Pongslip 2012; Tesfahunegn and Gebru 2020). Apart from using serology to study the diversity of nodulating bacteria, its practical relevance is to identify strains that are vital in managing symbiosis. Despite many studies documenting serological diversity within nodule bacteria populations, relatively few authors have exploited these variations to predict symbiotic performance (Kawaka 2016; Kawaka et al. 2018; Vitorino and Bessa 2017). The use of serology to classify bacteria has weaknesses such as presence of strains that are un-reactive against all antisera tested (Remigi et al. 2016), non-reactive strains and cross-reaction of strains with antiserum derived from reference strains (Kawaka et al. 2018; Zhang et al. 2014).

## Antibiotic resistance

Microbial studies in natural habitats require recovery of either the resident population or added cell on selective media that excludes other contaminants in the environment. The absence of suitable media that allows for selective recovery of symbiotic bacteria in soil has hampered studies on the behavior of these bacteria (Ondieki et al. 2017). Symbiotic bacteria like other bacteria consist of few naturally occurring mutants that are tolerant to high concentrations of selected antibiotics (Naamala et al. 2016). Growing of selected antibiotic resistant mutants in media that contains elevated levels of anti-microbial agents has been used to identify symbiotic bacterial strains and other bacteria (Spriggs and Dakora 2009). Culturing these bacteria on YEMA plates containing antibiotic markers target symbiotic strains with resistant strains retaining their biological nitrogen fixation abilities (Kawaka et al. 2018; Mora et al. 2014). The antibiotic resistant marked strains are identified by the fact that they can grow on media containing the antibiotics while non-marked ones are unable to grow (Knight et al. 2019). The technique is preferred when strain identification by serology is not possible as a result of cross reaction of strains or due to lack of antisera. The technique is popular due to the ease of obtaining mutants resistant to streptomycin (Baldani et al. 2014; Fair and Tor 2014).

Usually the mutant strain will grow on the antibiotic media and other bacteria will be suppressed (Enne et al. 2006). It is crucial to ensure that antibiotic-resistant mutants that are selected for inoculation experiments have not lost their ability to form nodules or their ability to fix nitrogen with the host plant. Symbiotic capacity of the mutant is always compared with its parent culture from time to time (Voisin et al. 2015). The mutant should also be stable throughout the steps of infection, nodulation, nitrogen-fixation and subsequent re-isolation.

## 16S rRNA gene sequence

Earlier taxonomic studies described rRNA gene as an ideal marker for bacterial phylogenetic analysis (Gornung 2013; Idris et al. 2020). Sequencing the 16S rRNA gene is considered as a model genetic marker for classifying and identifying bacterial species including symbionts (Caputo et al. 2019). The gene sequence analysis is efficient in classifying poorly described (Clarridge 2004), rarely isolated (Fredricks and Relman, 1996), phenotypically aberrant strains and identification of novel noncultured bacteria (Clarridge 2004; Stöhr et al. 2005).

**Fig. 1:**
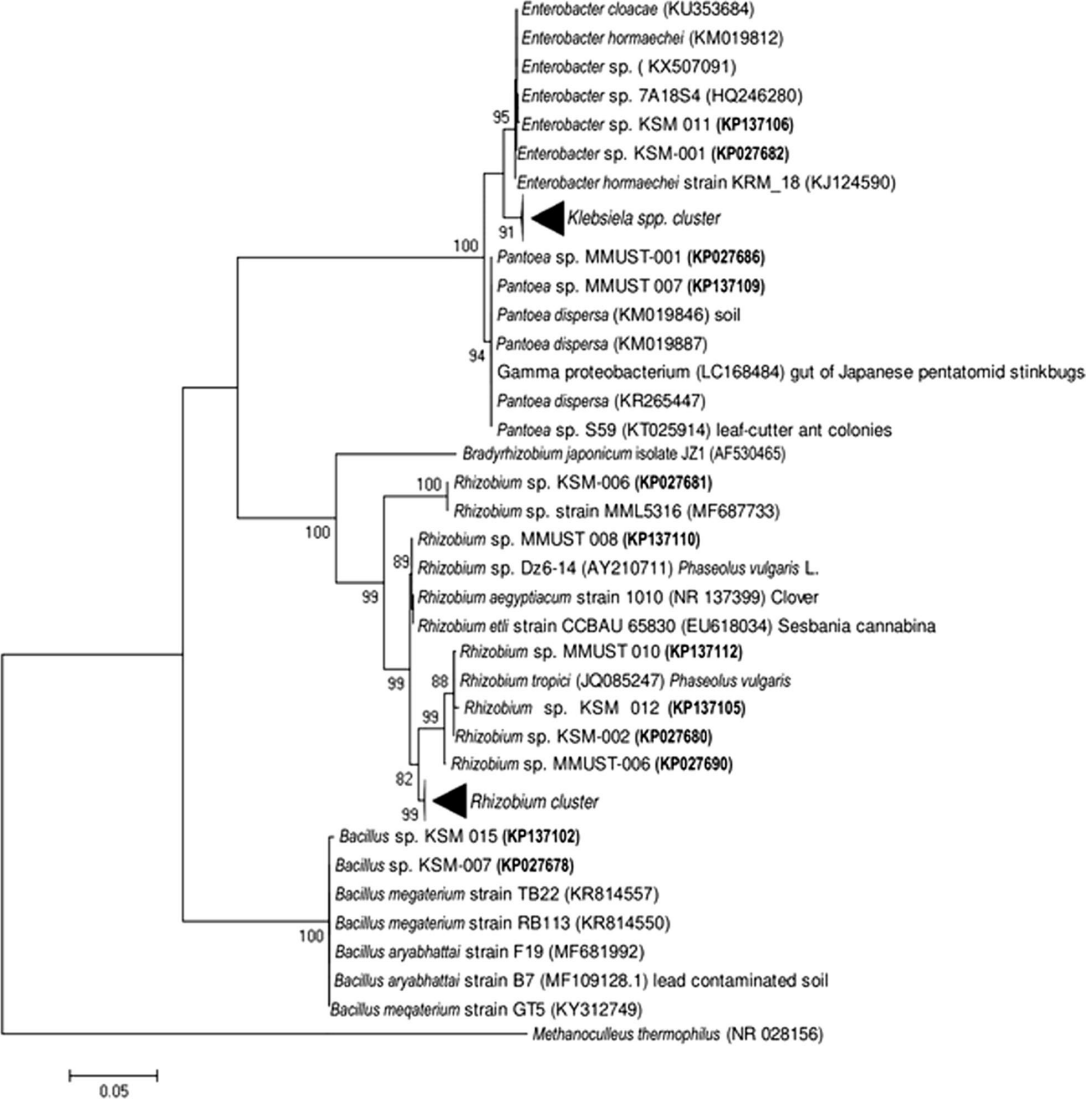
Phylogenetic tree of the 16S rRNA gene isolates (in bold) and closely affiliated species

The rRNAs form important parts of ribosomes that that are needed in mRNA translation (Acinas et al. 2004). This genetic marker has features making it the preferred technique for phylogenetic analysis. Firstly, 16S rRNA gene is found in all organisms thus allows comparison of genetic relationship among organisms through phylogenetic tree analysis. Secondly, the gene is highly conserved and does not change over time demonstrating that random sequence variations in organisms can provide a precise measure of evolution. The level of conservation in 16S rRNA gene results from its vital role as a key component in the cell compared to other genes like those required for enzyme synthesis. Mutations in enzyme genes are frequently tolerated because they interfere with structures not essential as the rRNA gene. Lastly, 16S rRNA gene sequence is approximately 1500 bp in length including the conserved and variable regions that provide sufficient information required for taxonomy. Usually, conserved regions are important in designing primers and allow alignment of sequences of organisms that are remotely related (Chakravorty et al. 2007). Following these advantages, 16S rRNA gene sequence is widely preferred as a technique for phylogenetic classification of symbiotic bacteria (Janda and Abbott 2007). Generally, the similarity of 16S rRNA gene sequences is an important threshold for delineation of many species. Consequently, a large number of 16S rRNA gene sequences are available in the nucleotide databases. Based on the sequences availability in the database for comparison, researchers consider 16S rRNA gene as the preferred marker for identification and constructing phylogenies (Fuks et al. 2018). Construction of a phylogenetic tree using 16S rRNA has revealed close taxonomic affiliation of symbiotic bacteria species from diverse soils as shown in Fig. 1 (Kawaka et al. 2018). The generation of phylogenetic trees uses morphological, biochemical, behavioral or molecular features of species or other group. As indicated in the Fig. 1, trees depict lines of evolutionary descent of different species, organisms or genes from a common ancestor. Phylogenies are useful for structuring classifications and provide insight into events that occurred during evolution. In addition, trees show descent from a common ancestor and therefore it is crucial to understand phylogenies in order to fully appreciate evidence supporting the theory of evolution.

However sequences in certain databases are not regularly updated and accurate leading to lack of consensus on the reliability of 16S rRNA gene sequence data (Clarridge 2004; Woo et al. 2008). Notwithstanding, the accuracy of 16S rRNA gene analysis is not widely used and only restricted to large and reference laboratories due to technical expertise and high cost (Clarridge 2004; Woo et al. 2008).

## DNA-DNA hybridization

DNA–DNA hybridization (DDH) is a common technique for analyzing genomic similarity to determine bacterial taxonomy (Auch et al. 2010; Degefu et al. 2013). The technique is used as tool in determining specific variations among closely related microbial species. The method has enjoyed an enormous relevance since 1960s as a regular criterion for describing new bacterial species including symbionts (Krieg, 1988). The concept is based on the ability of hybridized DNA of related organisms to withstand thermal variation and entire similarity is calculated from pairwise whole genome comparisons (Markegard et al. 2016; Rollinson and Stothard 2017).

The DNA molecule is denatured then returns to its original conformation by reducing the temperature, which is referred to as a reassociation temperature and involves three steps (Wang et al. 2014). Firstly, shearing of the genomic DNAs of assayed unknown organism and reference strain into small fragments of about 600–800 bp (Fitzgerald et al. 2015). Secondly, the mixed DNA fragments of the two strains are heated to dissociate the double strands (Auch et al. 2010) and finally cooling the temperature down until the fragments re-anneal (Wang et al. 2014). The level of matching base pairings of the two strands depends on the melting temperature of double strands (Auch et al. 2010; Wang et al. 2014). The genomic relatedness of two strains is estimated from the melting temperature (Gasser et al. 2008). Usually, DDH value ≤ 70% is considered as an indication that the unknown bacteria are different from the reference strain (Tindall et al. 2010; Wayne et al., 1987). Despite estimating similarity between genomes, DDH is a tedious, inaccurate, error prone and gives conflicting results (Rosselló-Mora 2006). The method provides non-cumulative relative DNA similarity values and therefore it cannot be used to set up incrementally comparative database (Rosselló-Mora 2006). DDH technique requires a large amount of quality DNA, technical expertise, specialized laboratories and applied only on strains that have gene sequences (Tindall et al. 2010). Due to the current developments of genome sequencing, DDH method is likely to be replaced by alternative techniques based on genome sequence comparisons (Du et al. 2013; Oren 2004). Until costs associated with sequencing are reduced, DDH still remains the method of choice to genomically delineate species.

In the recent past, the use of multiple protein-encoding housekeeping genes has gained a wide usage as a tool for investigating taxonomic relationships (Uelze et al. 2020). Housekeeping genes was proposed as a portable sequence-based method for identifying clonal relationships among bacteria. The method uses information from multiple genes to give an overall and reliable relationship among organisms. Sequencing of at least five housekeeping genes that are universally distributed as single copies and located at distinct chromosomal loci offers a great promise for bacterial taxonomy. Compared to other taxonomic methods such as 16S rRNA genes, the higher degree of sequence divergence of housekeeping genes is superior for identification purposes. The more conserved 16S rRNA gene sequences do not always allow species discrimination (Ferraz Helene et al. 2022; Haque et al. 2017). In addition, a small number of carefully selected gene sequences could be equal or even surpass the precision of DNA–DNA hybridization for quantification of genome relatedness (Haque et al. 2017). Housekeeping genes yields sequence clusters at a wide range of taxonomic levels ranging from intraspecific through the species level to clusters at higher levels (Leray et al. 2019). Together with DNA-DNA hybridization, analysis of housekeeping genes has the potential be considered as a standard practice in bacterial taxonomy.

## Whole genome sequencing

Whole Genome Sequencing (WGS) is a technique that analyses the entire chromosomal DNA of an organism and the DNA of mitochondria, chloroplast or plasmids at a single time. WGS is the most informative and comprehensive method of characterizing genomes.

WGS allows the inference of the phylogenetic relationship between a set of bacterial strains. The technique is very appealing and enables the identification of additional classes of mutation that are refractory to detection by exome sequencing. WGS offers the opportunity to interrogate noncoding regions of DNA and identify functionally important sequence variants that influence gene expression.

Currently, researchers have sequenced a large number of bacterial genome and the data is easily accessed from public nucleotide databases such as the Genebank (Land et al. 2015). The technology is increasingly being adopted in classifying nitrogen-fixing and related bacteria (Uelze et al. 2020). So far complete genome sequences of *Rhizobium*, *Sinorhizobium*, *Mesorhizobium*, *Bradyrhizobium* and *Azorhizobium* among others have been sequenced and available for public use (Molina-Sánchez et al. 2015; Sugawara et al. 2013). The technique provides complete genetic variation and the sequence data can be used for identification and taxonomy of organisms.

Due to the rapid drop in the price of technology, it is projected that many more symbiotic bacteria complete genomes will be sequenced. Sequencing whole genome is still expensive as it requires specialized laboratories and skilled expertise to analyze the sequence data. Researchers still use nucleotide sequences of different genes and genetic fingerprints for phylogenetic and diversity studies despite the markers having limited molecular information. As the cost of sequencing continues to decrease and experience is gained in data analysis and interpretation, it is anticipated that WGS will be the method of choice for future research.

## Metagenomics

The technique involves genomic analysis of microorganisms by direct extraction and cloning of DNA from an assemblage of microorganisms. Development of metagenomics stems from the inevitable evidence that uncultured microorganisms represent the vast majority of organisms in most environments. The evidence arise from analyses of 16S rRNA gene sequences amplified directly from the environment, this approach avoided the bias caused by culturing and eventually led to the identification of new microbial lineages (Bowers et al. 2021). The microbial world has been revolutionized by analysis of 16S rRNA genes however such studies have yielded only a phylogenetic description with little insight into the genetics, physiology, and biochemistry of the members. The use of metagenomics has provided a second tier of technical innovation that facilitates study of the physiology and ecology of environmental microorganisms (Lear et al. 2021). Metagenomics has led to the discovery of novel genes and gene products including the first bacteriorhodopsin of bacterial origin, novel molecules with antimicrobial activity and new proteins, RecA, DNA polymerase, and antibiotic resistance determinants (Kwon et al. 2013). The reassembly of multiple genomes provides an insight into energy and nutrient cycling, genome structure, gene function, population genetics and microheterogeneity and lateral gene transfer among members of an uncultured community (Handelsman 2004). Utilization of metagenomic sequence information has the potential to facilitate the design of better culturing strategies to link genomic analysis with pure culture studies. Metagenomics has redefined the concept of a genome and accelerated the rate of discovery of new genes. The technique has been widely used in biotechnology to screen functional enzymes, antibiotics and many reagents in libraries from different environments (Popovic et al. 2017). However, quite a number of barriers have impeded the discovery of new genes that could be used to solve medical, agricultural, or industrial problems.

Metagenomics is also considered the primary technique for studying phylogeny and taxonomy of complex microbiomes (Berg et al. 2020). Microbiome research has evolved rapidly over the past few years however their phylogeny and taxonomy are more complex and less studied (Meisner et al. 2022)*.* The use of metagenomics has significantly enhanced understanding on metabolic, physiological and ecological roles of environmental microorganisms (Strazzulli et al. 2017). However, analysis of the microbiome is affected by experimental conditions such as sequencing errors and genomic repeats (Berg et al. 2020). Furthermore, the introduction of new sequencing technologies and protocols has led to numerous new methodologies that negatively affect results of the analyses. There are several specific marker/target genes that have been identified for studying microbiome (Meisner et al. 2022). These marker genes are functionally conserved across phylogenetic distances thus serving as a molecular clock for studying evolutionary changes. The highly conserved 16S rRNA gene has a crucial cellular role and survival forming the basis of obtaining precise genomic classification of known and unknown microbial taxa.

## Comparative proteomics

Proteomics is a high-throughput technology that has been adopted to investigate a wide range of biological aspects including phylogenetic and molecular divergence studies.

In the recent past, considerable attempts have been made to characterize the diversity of proteins expressed in different tissues under a variety of conditions (Faize et al. 2020; Kawaka et al. 2018). The prospect of identifying bacteria using mass spectrometry (MS) and its role in detection and characterization of microorganisms has elaborately been described (Rahi and Vaishampayan 2020). Initially, mass spectrometry was introduced to rapidly identify intact microorganisms (Nomura et al. 2020). Following the development of proteomics and bioinformatics, protein databases have successfully supported MS identification of microorganisms. Using 50 subunit ribosomal proteins, many bacterial species have been identified (Tatsukami and Ueda 2016). Similarly, matrix-assisted laser desorption/ ionization time-of-flight MS (MALDI TOF MS) correctly identified 408 and 360 g-negative bacilli strains at the genus and species levels at a successful rate of 93% and 82% respectively (Jia et al. 2015).

Recently, MS technique for rapid identification and classification of microorganisms has attracted great interests from microbiologists for use in symbiotic bacteria research (Vitorino and Bessa 2017). For example, MALDI TOF MS showed a fast and reliable platform for identification and ecological studies of species from the family *Rhizobiaceae* (Ashfaq et al. 2022). MALDI TOF MS has also been applied for in situ identification of plant invasive nodule bacteria in different legumes (Ziegler et al. 2012). Nonetheless, the MALDI TOF MS technique requires a well-established reference spectral database for accurate bacterial identification. Sample preparation and period of growth of bacteria such as symbiotic bacteria affects the quality and reproducibility of the protein mass spectra (Mandal et al. 2007).

## Polyphasic taxonomy

Polyphasic taxonomic approach puts emphasis the use of classical methods in combination with modern genetic/molecular techniques for bacterial delineation (Chan et al. 2012). The method takes into account all available phenotypic and genotypic data and integrates them into a consensus type of classification. The classical techniques such as morphological and biochemical descriptions are usually used as well as chemotaxonomic features like cell wall, polar lipid, fatty acid, and respiratory menoquinones (Yadav et al. 2020). These crucial diagnostic biomarkers help in the general assignment of isolates to their correct taxa. Chemotaxonomic characteristics are useful in reflecting phylogenies at the genus/family level. Modern molecular techniques focus on variable and conserved regions that are assessed by comparing multiple sequence alignments and viewed as phylogenetic trees (Chowdhury and Garai 2017). However these taxonomic classifications do not necessarily define the expected physiological traits since closely related organisms from different locations can have very distinct physiologies and metabolic processes. Therefore, it is essential to conduct laboratory investigations on the isolates from different regions and compare to reference strains of closely related organisms. Despite modern molecular techniques revolutionizing bacterial taxonomy, they still require reinforcement by chemotaxonomic and biochemical considerations. Different studies have shown that a combination of descriptive classical techniques together with modern molecular sequencing methods has resulted in precise identification of new taxa (Berg et al. 2020; Hyde et al. 2019). In future, to improve the effectiveness of polyphasic taxonomic approach, there needs to be a collaborative effort by specialized laboratories to guarantee a more stable consensus on bacterial classification. Otherwise, the technique will have to cope with challenges such as enormous amounts of data, large numbers of strains and data fusion which will require efficient centralized data storage.

## Conclusions

There has been an increase in the number of tools for determining the identity and diversity of microbial samples in the last decades. This review has demonstrated that methods used in taxonomy have their own discriminating power varying from the individual or species levels to the genus, family and higher levels. The techniques further depend on the field of application, particular conditions, the number and the type of strains. The degree of discrimination of a technique may vary and depends on the target bacterial taxon. It is therefore important to adopt the use of a technique with minimal contradictions that emphasizes fast and reliable features for identification.

However, phenotypic and cultural techniques remain the preferred presumptive methods of classifying symbiotic bacteria despite their limitations and challenges. Development of new molecular tools has really improved the identification of new legume bacteria and discovery of elite species that are effective in biological nitrogen fixation. Using an appropriate and informative technique, it is possible to correctly identify novel bacterial species with superior nitrogen fixing abilities. These strains would be vital in developing inoculation programs and boost legume production especially in developing countries facing food and nutrition insecurities under changing climatic conditions.

## Data Availability

Not applicable.
